# Mesenchymal Stromal Cells’ Therapy for Polyglutamine Disorders: Where Do We Stand and Where Should We Go?

**DOI:** 10.3389/fncel.2020.584277

**Published:** 2020-10-06

**Authors:** Inês Barros, Adriana Marcelo, Teresa P. Silva, João Barata, David Rufino-Ramos, Luís Pereira de Almeida, Catarina O. Miranda

**Affiliations:** ^1^CNC—Center for Neuroscience and Cell Biology, University of Coimbra, Coimbra, Portugal; ^2^CIBB—Center for Innovative Biomedicine and Biotechnology, University of Coimbra, Coimbra, Portugal; ^3^III—Institute for Interdisciplinary Research, University of Coimbra, Coimbra, Portugal; ^4^Faculty of Pharmacy, University of Coimbra, Coimbra, Portugal; ^5^Viravector—Viral Vector for Gene Transfer Core Facility, University of Coimbra, Coimbra, Portugal

**Keywords:** mesenchymal stromal cells, polyglutamine disorders, neurodegenerative disorders, cell therapy, extracellular vesicles, secretome

## Abstract

Polyglutamine (polyQ) diseases are a group of inherited neurodegenerative disorders caused by the expansion of the cytosine-adenine-guanine (CAG) repeat. This mutation encodes extended glutamine (Q) tract in the disease protein, resulting in the alteration of its conformation/physiological role and in the formation of toxic fragments/aggregates of the protein. This group of heterogeneous disorders shares common molecular mechanisms, which opens the possibility to develop a pan therapeutic approach. Vast efforts have been made to develop strategies to alleviate disease symptoms. Nonetheless, there is still no therapy that can cure or effectively delay disease progression of any of these disorders. Mesenchymal stromal cells (MSC) are promising tools for the treatment of polyQ disorders, promoting protection, tissue regeneration, and/or modulation of the immune system in animal models. Accordingly, data collected from clinical trials have so far demonstrated that transplantation of MSC is safe and delays the progression of some polyQ disorders for some time. However, to achieve sustained phenotypic amelioration in clinics, several treatments may be necessary. Therefore, efforts to develop new strategies to improve MSC’s therapeutic outcomes have been emerging. In this review article, we discuss the current treatments and strategies used to reduce polyQ symptoms and major pre-clinical and clinical achievements obtained with MSC transplantation as well as remaining flaws that need to be overcome. The requirement to cross the blood-brain-barrier (BBB), together with a short rate of cell engraftment in the lesioned area and low survival of MSC in a pathophysiological context upon transplantation may contribute to the transient therapeutic effects. We also review methods like pre-conditioning or genetic engineering of MSC that can be used to increase MSC survival *in vivo*, cellular-free approaches—i.e., MSC-conditioned medium (CM) or MSC-derived extracellular vesicles (EVs) as a way of possibly replacing the use of MSC and methods required to standardize the potential of MSC/MSC-derived products. These are fundamental questions that need to be addressed to obtain maximum MSC performance in polyQ diseases and therefore increase clinical benefits.

## Introduction

Polyglutamine (polyQ) diseases are a group of nine inherited neurodegenerative disorders including dentatorubral pallidoluysian atrophy, spinal bulbar muscular atrophy (SBMA), Huntington’s disease (HD), and spinocerebellar ataxias (SCAs) type 1, 2, 3, 6, 7 and 17. All of them are autosomal dominant, apart from SBMA, which is X-linked (Orr and Zoghbi, 2007).

PolyQ disorders are associated with the unstable expansion of the cytosine-adenine-guanine (CAG) trinucleotide repeat in the respective causative gene. Such mutation encodes for extended glutamine (Q) tract, which occurs in different proteins according to the disorder, thus having unrelated functions and distinct cellular and subcellular locations. Nonetheless, all polyQ diseases share common features suggesting that the polyQ stretch directly contributes to the toxic properties of these proteins through a “toxic gain of function.”

Although these disorders express distinct symptoms, all of them greatly impact a patient’s quality of life by leading to both physical and psychological complications. Some of the shared common features include: onset typically during midlife; the inverse correlation between the number of repeats and the age of onset; degeneration of specific neuronal subpopulations that slowly progress over 10–20 years after the onset, culminating in premature death (Maciel et al., 1995; Ranum et al., 1995; Dürr et al., 1996; Zoghbi and Orr, 2000). The cerebellum, basal ganglia, brainstem nuclei, and spinal motor nuclei are some of the regions of the nervous system that are transversely affected in polyQ diseases (Zoghbi and Orr, 2000; Ross et al., 2003). The neuropathological alterations are the result of cellular changes such as accumulation of polyQ proteins in the cytoplasm and nucleus, the formation of inclusions, disturbance of the quality control systems of the cell, mitochondrial dysfunction, axonal transport disruption, synaptic activity decline, and neuroinflammation (Shao and Diamond, 2007; Havel et al., 2009). Additionally, the generation of toxic fragments resulting from the proteolytic cleavage has also been described in a few polyQs. The existence of common pathological mechanisms opens windows for the design of possible common therapies. In that sense, cell therapies and cellular-derived treatments are good candidates to be used for that purpose.

Currently, besides palliative care, treatments for polyQ disorders are only able to suppress or reduce specific symptoms. Levodopa or a dopamine agonist have been successful in cases displaying parkinsonian features, with dystonia and bradykinesia also being reduced through this approach (Tuite et al., 1995; Paulson, 2007). Furthermore, gait symptoms plus dysarthria can be improved by occupational and physical therapy, and dysphagia might be ameliorated by implementing strategies on what and how patients eat to avoid complications (Paulson, 2007). On the other hand, since pre-symptomatic testing already exists, medical and ethical guidance regarding treatment and parenthood counseling can be given, not only to post-symptomatic patients but also to pre-symptomatic individuals (Sequeiros et al., 1998; Drüsedau et al., 2004; Bettencourt and Lima, 2011; Schuler-Faccini et al., 2014).

Despite the efforts that have been made to develop effective therapies, there is still no strategies that can prevent, cure, or delay the progression of polyQ disorders.

Promising molecular and cellular therapies have been investigated in the last decades. Regarding gene therapy, the most frequently used method consists of silencing the defective protein resulting from the CAG expansion in a given gene (depending on the polyQ), mostly using RNA interference. However, there are challenges regarding delivery to the central nervous system and the risk of off-target effects. Significant progress has been made regarding this approach and such strategies will probably be implemented soon. Still, since the genetic defects differ from disorder to disorder, the strategy will require adaptations for each disease. As this subject is not the main focus of this review, we will not discuss it in detail, but this subject is reviewed in Matos et al. (2018).

Cellular therapies, on the other hand, may exert a pan effect on the common defective mechanisms that polyQ disorders share, as described above. For that, neural precursor and neural stem cells, embryonic stem cells, induced pluripotent stem cells, and mesenchymal stem cells (MSC) can be envisioned. Naturally, each source of the referred stem cells has its advantages, drawbacks, and success upon usage in polyQ diseases, as summarized elsewhere (Mendonça et al., 2018). Presently, MSC is not the first choice for cellular therapies in neurodegenerative conditions. Nonetheless, they are undoubtedly the safest kind among the different stem cells that can be used for therapeutic purposes since they are adult cells, and thus, not prone to develop tumors in a stable organism. Therefore, they have been widely used in studies of neurodegenerative disorders, including polyQs. However, despite some promising results, some aspects need to be improved to amplify MSCs healing effects and achieve translational significance. These concerns will be discussed in the present review article.

## Mesenchymal Stromal Cells (MSC): Promising Tools for The Treatment of PolyQ Disorders?

**Figure 1:**
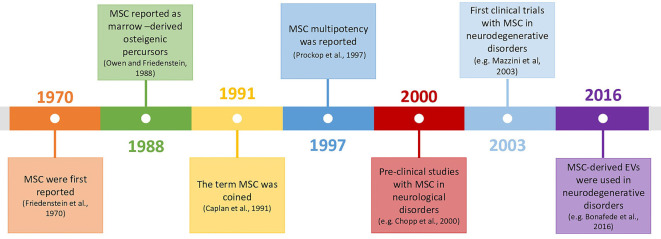
Timeline of mesenchymal stromal cells (MSC)’s history and their use in neurodegenerative disorders (Friedenstein et al., 1970; Owen and Friedenstein, 1988; Caplan, 1991; Prockop, 1997; Chopp et al., 2000; Mazzini et al., 2003; Bonafede et al., 2016).

Mesenchymal stromal cells (MSC) are adult multipotent progenitor cells capable of giving rise to tissues from the mesenchymal lineage. They were described in the 70’s decade by Friedenstein et al. (1970, 1974) and since the discovery of their multipotency in 1997 (Prockop, 1997), their use in cellular therapies for neurodegenerative disorders suffered a steadfast evolution, due to the high potential/low-risk balance (Figure 1).

**Figure 2:**
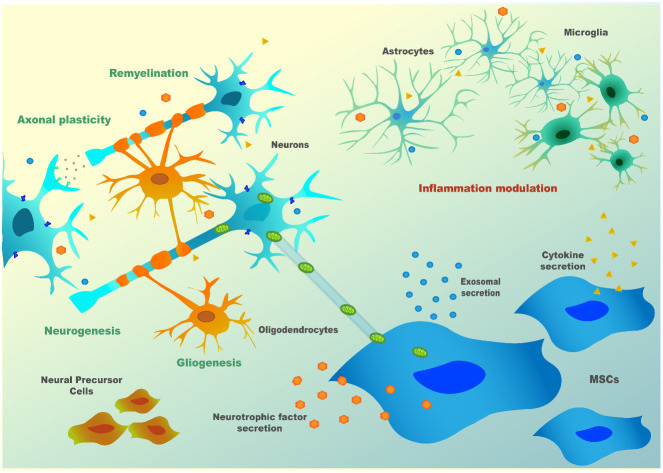
MSC’s paracrine mechanism(s) in neuronal cells.

In neuronal context, their role was initially thought to be associated with the replacement of aged and damaged cells (Wakitani et al., 1995; Pittenger et al., 1999) but, currently, there is consensus that this is not their principal mode of action. Cell fusion was also described to occur by some authors. Of note, this mechanism was described in a SCA1 mouse model, where Purkinje cells (PCs) of the host animals fused with MSC (Huda et al., 2016). However, this mechanism is rare in the literature and does not appear to account significantly for MSC’s overall outcomes. So MSC’s main effects are probably mainly mediated by influencing neighboring cells, through secretion of bioactive factors, or by inducing secretion of these factors in host cells, thus being able to modulate the immune system and to promote tissue repair (Gao et al., 2001; Tremain et al., 2001). The establishment of strong communication through chemical signals between MSC and surrounding cells, which can be either other MSC, neurons, astrocytes, or glia, is thought to be the anchor for MSC’s therapeutic capabilities. Indeed, MSC can respond to stress “signals” of the nearby cell by modulating their molecular pathways towards the production of specific factors, which can support these cells under stress (Millard and Fisk, 2013). As an example, a mechanism called “bio bridge” in which MSC seem to form a stream of extracellular matrix (bridge) between the lesion site and neurogenic niches was described for some neurological contexts such as traumatic brain injury and stroke, and could occur in other neurological disorders (Tajiri et al., 2013; Sullivan et al., 2015). This bridge is concomitant with higher expression levels of metalloproteinases (MMP) such as MMP9, leading to better communication between MSC and endogenous neuronal precursors and increment in neurogenesis post-lesion. Precursors cells from neurogenic niches then reach the brain-lesioned tissue, replacing injured cells. For all these mechanisms, there is evidence of bidirectional communication exerted through bioactive soluble molecules [neurotrophins, cytokines, chemokines, microRNAs (miRNAs), etc.], extracellular vesicles (EVs; that can contain proteins, RNAs, or even DNA) or by direct contact (e.g., *via* tunneling nanotubes or through mechanotransduction). Therefore, depending on the defects in the host damaged tissue, MSC may: (1) modulate inflammatory processes; (2) reduce oxidative stress, either by inducing survival pathways or by the direct transfer of healthy mitochondria to the host cells (*via* nanotubes); (3) favor neurogenesis by the secretion of neurotrophins and by the formation of “bio bridges”; (4) induce gliogenesis and remyelination; and (5) increase axonal survival and plasticity, thus inducing synaptogenesis (Paul and Anisimov, 2013; Figure 2). These exquisite cross-talks lead to a wide evaluation of MSC for the therapy of neurological diseases in preclinical and clinical models.

From Alzheimer’s (AD) to Parkinson’s (PD) or HD, the encouraging effects of MSC in a few pre-clinical studies prompted clinicians to perform preliminary clinical trials to evaluate their safety and/or effectiveness. However, this process started before fundamental issues were properly addressed at the pre-clinical level, which led to some disappointing results relative to the ones expected. Due to the initial lack of information, strategies did not contemplate solutions for problems such as the challenge of surpassing the blood-brain barrier (BBB), the low rate of cells engraftment in the lesioned tissue, the low survival of MSC, or the unidentified mechanisms involved in MSC’s positive effects. Finally, the standardization of MSC source of cells or even of methods capable of evaluating their potential, are imperative to make translation possible.

The investigation in this field is therefore currently aiming at resolving these difficulties and giving answers to the urgent need of efficacious therapies for neurodegenerative disorders for which therapeutic tools are presently scarce.

This review gives an overview of this subject with a particular focus on polyQ disorders, which besides HD, are scarcely referred to in the literature.

### Pre-clinical Studies Assessing MSC’s Therapeutic Potential in PolyQs

Several pre-clinical studies have investigated the therapeutic efficiency of MSC isolated from different sources, including bone marrow (BM-MSC), adipose tissue (AD-MSC), and umbilical cord (UC-MSC), in rodent models of HD. HD is the polyQ disease with the highest prevalence worldwide affecting about 1 in 7,500 individuals (Evans et al., 2013; Fisher and Hayden, 2014). HD causes brain atrophy in several regions such as the striatum, thalamus, cerebellum, brain stem, and cortex (Harper et al., 2005; Hassel et al., 2008; Labbadia and Morimoto, 2013; Chao et al., 2017) leading to progressive motor dysfunction and incoordination, cognitive impairment and psychiatric symptoms.

Over the last decade, it has been demonstrated that MSC can relieve phenotype and neuropathology of HD in both transgenic (Lee et al., 2009; Im et al., 2010; Snyder et al., 2010; Lin et al., 2011; Yu-Taeger et al., 2019) and chemically-induced models (Lee et al., 2009; Edalatmanesh et al., 2011; Rossignol et al., 2011, 2015; Hosseini et al., 2015; Ebrahimi et al., 2018). These studies show that animals treated with MSC displayed improved behavioral performance, cognitive functions, and, in the excitotoxic Quinolinic Acid (QA)-induced HD rats, reduction of apomorphine-induced rotation. Hosseini et al. (2015) also showed that MSC was able to reduce anxiety levels in treated QA-induced HD rats. Also, the administration of MSC was able to increase the survival of the R6/2 mouse model (Lee et al., 2009; Lin et al., 2011; Yu-Taeger et al., 2019). Importantly, one of these studies pointed out the importance of well-dosing the number of MSC administered. The authors compared two different doses of MSC (2 × 10^5^ and 4 × 10^5^) and surprisingly only the group treated with the lowest dose presented motor improvements (Rossignol et al., 2011). The authors concluded that a high number of MSC may cause tissue damage to striatal architecture.

Regarding neuropathological improvement, MSC preserved the volume of the striatum, induced neurogenesis, and differentiation of the host endogenous neural stem cells, and reduced neuronal death (Snyder et al., 2010). Lin et al. (2011) saw a decrease in the levels of Bax and caspase-3 as well as an up-regulation in the expression of Erk1/2, which suggests that MSC can inhibit apoptosis and promote neuronal survival. Furthermore, this study points out the strong capability of MSC for recruiting microglia, neuroblasts, and bone marrow-derived cells to the lesion site which helps tissue regeneration (Lin et al., 2011). MSC administration has also led to the reduction of the number of huntingtin aggregates (Lee et al., 2009).

Notably, Yu-Taeger et al. (2019) showed that MSC could ameliorate disruptions in the dopaminergic signaling cascade, increasing the expression of DARPP-32 and tyrosine hydroxylase in the striatum, known to be reduced in R6/2 HD mice (Bibb et al., 2000). This study also evidenced the immunomodulatory potential of MSC as treated mice restored the expression of Iba1, and microglia morphology in the striatum, plus inducing the downregulation of several inflammatory modulators (Yu-Taeger et al., 2019).

The feasibility of performing autologous transplants in HD has also been assessed. Researchers compared the efficacy of AD-MSC isolated from the subcutaneous adipose tissue of an HD patient and a healthy donor in the treatment of YAC128 transgenic mice, a long-lived HD model (Im et al., 2010). Even though both kinds of cells expressed the same cell surface markers and showed a similar secretory potential in terms of neurotrophic factors, normal AD-MSC could reduce striatal atrophy when they were transplanted into 8 month-old YAC128 mice, whereas HD AD-MSC failed to promote such benefits. Nonetheless, the injection of cells in this time-window did not produce improvements in any of the treated groups regarding rotarod performance or body weight. On the contrary, when transplanting normal AD-MSC at 12 months of age, there were significant improvements in motor performance. These results show that AD-MSC could modify the course of HD in long-lived mice, though their effect is dependent on the donor condition and time window of delivery.

More recently, two studies assessing how the number of passages of MSC in *ex vivo* cultures can affect their therapeutic effects were performed in R6/2 transgenic mice (Fink et al., 2013; Rossignol et al., 2015). In the first study, BM-MSC isolated from mice were grown for a low (3–8) or high (40–50) number of passages, and no neuronal or glial differentiation was observed. The striatum of R6/2 mice transplanted with BM-MSC at higher passage showed increased metabolic activity compared with non-treated R6/2 mice and significantly-improved behavioral sparing compared to either untreated R6/2 mice or R6/2 mice receiving low-passage BM-MSC. Thus, the passage number of BM-MSC expansion could alter their efficacy.

In the second study, they compared the therapeutic potential of low- (3–8) and high-passage (40–50) UC-MSC in the R6/2 transgenic HD mouse model (Fink et al., 2013). Interestingly, high-passage UC-MSC showed a higher survival rate *in vivo*, but contrary to the previous study, both transplanted groups showed increased metabolic activity and reduced brain damage in the striatal area. Moreover, no major differences were observed in the motor and non-motor behavioral tests compared with non-treated HD mice. In contrast, Ebrahimi and collaborators showed that the administration of UC-MSC at a low passage in 3-NP lesioned rat models led to significant improvement of motor performance and muscle activity. Plus, they also observed neuropathological ameliorations as UC-MSC were able to decrease gliosis and increase both the striatal volume and dendritic length of striatal cells (Ebrahimi et al., 2018). So, as a conclusion, the time of MSC expansion and concomitant number of passages is an important parameter and should be further and carefully addressed in future assessments.

The relief of phenotypical and neuropathological defects in HD models has been associated with MSC trophic effect. Several studies reported that MSC transplantation increased the expression of both messenger RNA (mRNA) and protein levels of brain-derived neurotrophic factor (BDNF) in the striatum (Snyder et al., 2010). Additionally, Snyder et al. (2010) reported that BM-MSC transplantation led to increased expression of the neurotrophic factors fibroblast growth factor (FGF), ciliary neurotrophic factor (CNTF), vascular endothelial growth factor (VEGF), and nerve growth factor (NGF). Over-expression of collagen type-I and fibronectin in the striatum of MSC-treated animals has also been observed which contributes to the stabilization of the extracellular matrix, helping tissue regeneration (Rossignol et al., 2011).

Regarding other polyQ disorders, few preclinical studies have been performed in mouse models of SCA1, SCA2, and SCA3. Despite the causative mutation being located in different genes, an expansion of the CAGs in *ATXN1*, *ATXN2*, and *ATXN3* (also known as *MJD1*), respectively, many symptoms and major mechanisms involved in disease progression are similar among SCAs (Paulson, 2009; Klockgether et al., 2019; Sullivan et al., 2019). Over time, patients develop motor incoordination as the main progressive clinical feature, along with tremors, muscle rigidity, dysarthria, dysphagia, peripheral neuropathy, oculomotor problems (such as double vision, nystagmus or ophthalmoplegia), and parkinsonism in some cases (Paulson, 2009). Cognitive impairments have also been pointed out to occur.

MSC’s effect has shown to be equally promising in these groups of polyQs, demonstrating that they can alleviate motor function deterioration in SCAs. A pre-symptomatic study was performed in a transgenic mouse model of SCA-1 (SCA1-Tg mice). Intrathecal (IT) injection of an MSC line (KUM10) in SCA1-Tg mice led to neuropathological improvements. MSC was shown to migrate towards the cerebellum and mitigate neuronal disorganization. The cerebellum of these SCA1 transgenic mice had ectopically-located PCs bodies that resulted in a multilayer of PC, and mice showed atrophy of the cerebellar molecular layer (ML). In the referred study, MSC-treated mice displayed a single layer of PC and the ML was thicker than that of the untreated mice. Motor function assessed by the rotarod test was also shown to be improved in treated animals (Matsuura et al., 2014).

Additionally, MSC showed to mitigate motor functions deterioration in a SCA2 transgenic mouse model (Chang et al., 2011). The study compared the efficacy of repeated intravenous (IV) or intracranial (IC) injections of human BM-MSC and concluded that IV infusions effectively delayed diseases’ onset and improved motor performance by rescuing cerebellar PC, whereas IC injections failed to improve motor skills. In this study, more grafted BM-MSC were found in the IV transplantation group, located in the cerebellum and cerebral cortex (CC). The authors explained the higher success of IV delivered BM-MSC due to the extravasation capacity as cells migrated from intracranial vessels through white matter (WM) into several regions of the cerebellum.

Our group has recently demonstrated that mouse BM-MSC transplantation can ameliorate both phenotypical and neuropathological MJD symptoms in Machado–Joseph disease (MJD)/SCA3 (Oliveira Miranda et al., 2018). In agreement with the previous study, our investigation showed that the administration of several consecutive IV injections of BM-MSC sustainably ameliorated motor impairments, when delivered post-symptomatically. Using intracerebroventricular administration (ICV), a single injection could only alleviate MJD symptoms transiently in the same Tg-ATXN3-69Q transgenic mouse model of MJD (Torashima et al., 2008). This suggests that repeated treatment through the IV route led to better outcomes. Notably, our results were in line with Li et al. (2018) findings, which showed that repeated systemic treatment of pre-symptomatic transgenic homozygous MJD mice [SCA3-YAC-84Q; (Cemal et al., 2002)] with human UC-MSC was able to alleviate motor impairments and neuropathology of MJD.

These findings were corroborated with low survival and poor engraftment in host tissues, which represent a common problem in these therapies. In our study, we demonstrated that after ICV transplantation, despite cells surviving up to 8 weeks after transplantation, the SPION-labeled MSC signal was drastically reduced from 1 to 8 weeks *in vivo*. Moreover, BM-MSC delivered IV was detected in the brain of mice 30 min after transplantation, but completely disappeared from circulation before completion of 7 days (Oliveira Miranda et al., 2018). Similarly, other studies in HD animal models also observed that MSC remained only for a short time in the lesion site (Snyder et al., 2010), or in other cases, although the authors could detect cells in the brain, only a small percentage of the implanted cells could survive (Lee et al., 2009; Im et al., 2010; Snyder et al., 2010; Lin et al., 2011; Yu-Taeger et al., 2019). A concise review of these studies is given in (Table 1).

**Table 1:** Sum up of the survival, grafting, and integration of mesenchymal stromal cells (MSC) in pre-clinical studies in animal models of polyglutamine (polyQ) disorders.

PolyQ	Study	Animal model	MSC’s source	N° of cells injected	Site of injection	Time of injection	Tracking time	Grafting sites	Integration and neuronal differentiation
HD	Bantubungi et al. (2008)	QA-induced rat striatal model	rat BM-MSC	1 × 10^5^	Unilateral injection in the striatum	1-week after the QA lesion	8 weeks after transplantation	1.65 ± 0.18 × 10^5^ BM-MSC found at the lesion site in the striatum at 3 and 8 weeks	BM-MSC remained undifferentiated
	Lee et al. (2009)	QA-induced rat striatal model	human AD-MSC	1 × 10^6^	Unilateral injection in the striatum	immediately after the QA injection	4 weeks after transplantation	AD-MSC found near the initial transplantation foci, forming a lump	Co-labeling with BDNF, calbindin, GABA, and GAD
		R6/2 transgenic mice*	human AD-MSC	0.5 × 10^6^	Bilateral injection in the striatum	8.5-weeks-old mice (post-symptomatic treatment)	4 weeks after transplantation	Few AD-MSC migrated toward the dorsolateral subventricular area, or into the striatum; mean survival rate: 13.5 ± 1.4% (*n* = 3)	low rate of *in vivo* proliferation of AD-MSC; expression of nestin (~70%), doublecortin (~80%), Tuj-1 (~25%), and GAD (~80%)
	Im et al. (2010)	YAC128 transgenic mice	human AD-MSC isolated from an HD patient and a healthy donor	5 × 10^5^	Bilateral injection in the striatum	8-months-old (pre-symptomatic) and 12-months-old (symptomatic)	4 weeks after transplantation	No cells detected extremely low % of cell survival AD-MSC found in the striatum and in the periventricular area	ND
	Snyder et al. (2010)	N171–82Q transgenic mice	human BM-MSC isolated from healthy donors	0.1 × 10^5^	Unilateral injection in the striatum	8-week-old mice (pre-symptomatic)	2–3 weeks after transplantation	Grafted BM-MSC do not survive long in the striatum (disappeared over 3–15 days); Only 15.1% survived in the first 24 h (15.063 ± 3.776)	BM-MSC did not proliferate, but induced neurogenesis and neural differentiation of the host endogenous neural stem cells
	Lin et al. (2011)	QA-induced mouse striatal model	Immortalized human BM-MSC	2 × 10^5^	Unilateral injections in the striatum	7 days after the QA lesion	8 weeks after transplantation	BM-MSC found in the striatum; a small amount of BM-MSC survived for 16 weeks	Some BM-MSC co-localized with GFAP, NeuN, and DARPP-32
		R6/2 transgenic mice	Immortalized human BM-MSC	2 × 10^5^	Unilateral injections in the striatum	12-week-old mice (post-symptomatic)	12 weeks after transplantation	BM-MSC found in the striatum 12 weeks after transplantation	Some BM-MSC differentiated: co-localization with DARPP-32, GFAP, and F4/80.
	Rossignol et al. (2011)	3NP-induced rat model	Rat BM-MSC	0.2 × 10^6^ or 0.4 × 10^6^	Bilateral injection in the striatum	28 days after the first administration of 3NTP	~10 weeks after transplantation	BM-MSC were visible in the implantation site	No neuronal differentiation of BM-MSC was observed
	Fink et al. (2013)	R6/2 transgenic mice	UC-MSC (from WT C57/BL6 mice pups)	0.4 × 10^5^ per hemisphere	Bilateral injection in the striatum	5-week-old mice (post-symptomatic)	6 weeks after transplantation	UC-MSC found in the striatum 6 weeks post-transplantation; Mice transplanted with high-passage UC-MSC showed more surviving cells than mice treated with low-passage UC-MSC	Barely any co-localization of UC-MSC with NeuN was found
	Hosseini et al. (2015)	QA-induced rat striatal model	Human AD-MSC purified from healthy male donors	2 × 10^5^	Unilateral injection in the striatum	7 days after the QA lesion	7 weeks	AD-MSC found in the brain	AD-MSCs far migrated into other brain regions (even into the cortex)
	Fink et al. (2013)	QA-induced rat cerebellar model	rat BM-MSC	2.5 × 10^5^	Injection into the cerebellum (folia VI)	48 h after the QA lesion	6 weeks after transplantation	BM-MSC found in the cerebellum; a few cells could migrate to deep layers	BM-MSC seemed to be integrating within host tissue
	Rossignol et al. (2015)	R6/2 transgenic mice	mouse BM-MSC	2 injections of 0.2 × 10^5^ (total of 0.4 × 10^5^)	Bilateral injection in the striatum	5-week-old mice (post-symptomatic)	6 weeks after transplantation	Both low-passage and high-passage BM-MSC were capable of surviving in the brain for up to the 6 weeks of following	No neuronal or glial differentiation was observed, as BM-MSC could not co-localize with the markers NeuN or GFAP.
	Ebrahimi et al. (2018)	3NP rat model	UC-MSC	2.5 × 10^5^	Bilateral injection in the striatum	7 days after 3-NP administration	1 month after injection	UC-MSC found in the striatum 30 days post-transplantation;	ND
	Yu-Taeger et al. (2019)	R6/2 transgenic Model	mouse BM-MSC	1 × 10^6^	Intranasal transplantation	4-week-old mice (post-symptomatic)	5 days and 7.5 weeks post-delivery of MSC	BM-MSC were found in the midbrain, striatum, and olfactory bulb 5 days post-transplantation At 7.5 weeks no signal was detected	ND
SCA1	Matsuura et al. (2014)	SCA1 transgenic mice (B05 line)	mouse BM-MSC	6 × 10^5^	Injection into the Meninges Covering the Cerebellum	5-week-old mice (early symptomatic)	1 h and 3 days after administration	1 h—BM-MSC were in between the meninges 3d— BM-MSC were essentially confined to the cerebellum in lobules 3, 4, 5, and 6 and in the spaces between the folia	ND
SCA2	Chang et al. (2011)	SCA2 transgenic mice	Human clonally derived BM-MSC	IV: 4.2 × 10^7^/kg	IV	IV: 12, 23, 33 and 42-week-old mice	IV: 8 weeks after the last injection	IV: BM-MSC found in the cerebellar WM, ML, and lumens of blood vessels in the WM. Large clusters of grafted hMSC were detected in the CC.	ND
		SCA2 transgenic mice	Human clonally derived BM-MSC	IC: 8.4 × 10^6^/kg	IC (through the foramen magnum, into the position of the cerebellum)	IC: 12, 23, and 33-week-old mice	IC: 17 weeks after the last injection	IC: BM-MSC not detected over cerebellar WM, ML, or PC layer, but limited to a few lumens of blood vessels and a few scattered cells in the CC.	ND
SCA3/MJD	Oliveira Miranda et al. (2018)	SCA3/MJD transgenic mice (Tg-ATXN3–69Q model)	mouse BM-MSC	ICV: 3 × 10^5^	ICV (unilateral injection in the lateral ventricle of the brain)	ICV: Unilateral injection in the lateral ventricle of the brain (4–7.5-week-old mice)	ICV: 7 weeks after administration	ICV: BM-MSC found in the lateral ventricles, but the volume of MSC graft drastically reduced from 1 to 4 weeks after injection (55% volume reduction)	ND
		SCA3/MJD transgenic mice (Tg-ATXN3–69Q model)	mouse BM-MSC	IV: 4.5–8 × 10^7^/Kg	IV	IV: every 2–3 weeks, four consecutive times; first treatment at 4–6.5-weeks-old	Iv: 7 days	IV: BM-MSC engrafted mainly in the lungs but could also reach the brain parenchyma however remained there just for the first hours.	ND
	Li et al. (2018)	(YAC transgenic MJD model, homozygous–84Q)	Human UC-MSC	2 × 10^6^	IV	Treatment started at 3-months-old mice (pre-symptomatic) and consisted of 2 × 10^6^/week, within 12 weeks	3 months	UC-MSC found in the pons, PCL, and ML of the cerebellum 3 months after transplantation	UC-MSC did not co-localize with NeuN, GFAP, or calbindin

*List of abbreviations: HD, Huntington’s disease; QA, quinolinic acid; BM-MSC, Bone marrow-derived mesenchymal stromal cells; AD-MSC, adipose tissue-derived mesenchymal stromal cells; BDNF, Brain-derived neurotrophic factor; GABA, gamma-aminobutyric acid; GAD, glutamic acid decarboxylase; YAC, yeast artificial chromosome; ND, not determined; GFAP, glial fibrillary acidic protein; NeuN, neuronal nuclei; DARP-32, dopamine- and cAMP-regulated neuronal phosphoprotein; F4/80, major macrophage marker; UC-MSC, umbilical cord-derived mesenchymal stromal cells; 3-NP, 3-nitropropionic acid; SCA1, Spinocerebellar ataxia type-1; SCA2, Spinocerebellar ataxia type-2; IV, Intravenous injections; IC, Intracerebral injection; WM, white matter; ML, molecular layer; PCL, Purkinje cell layer; CC, cerebral cortex; SCA3/MJD, Spinocerebellar ataxia type-3 or Machado-Joseph disease; ICV, Intracerebroventricular injection*.

Interestingly, these studies in SCAs also relate the action of MSC to neuronal survival/activity pathways, which is following the previous studies performed in HD rodent models. In the study of Li et al. (2018), the neuroprotective action of MSC was linked with the upregulation of the insulin-like growth factor 1/heat shock protein 70 (IGF-1/HSP70) pathway. In our study, we demonstrated that MSC protected GABAergic and Glutamatergic neurons, as by proton magnetic resonance spectroscopy (1H-MRS) we detected an increased expression of Gaba and the complex Glutamine-Glutamate upon a treatment, along with increased levels of mRNA for the receptors Gabrb2 and Grm1 in the cerebellum, denoting a higher synaptic activity (Oliveira Miranda et al., 2018).

In conclusion, despite the general agreement on the fact that MSC promotes beneficial outcomes in polyQ diseases, the different severity grades of the rodent models used as well as the ability to simulate human diseases make the interpretation of the potency scale of the treatments difficult. The development of models that better recapitulate polyQ diseases neuropathology in humans would be essential. Thus, knock-in models of larger mammals like the HD CRISPR-Cas9 knock-in pig model that shows strong neurodegeneration patterns (Yan et al., 2018) could be an important contribution to the field to validate findings achieved with rodent models and test a possible replacement of degenerated neurons promoted by MSC.

Furthermore, different cell sources, heterogeneous methods of isolation, discrepancies in MSC origins (i.e., from healthy/disease tissues) and characteristics (cell passage number, conditions used during cell growth) have been used throughout the studies, which makes it difficult to reach a correct comprehension and make assertive conclusions with enough confidence to implement these therapies in the clinics.

### Clinical Studies in PolyQ Patients: Achievements and Flaws

Despite the extensive number of unanswered issues from the pre-clinical assessments herein referred, the promising results obtained from research on the use of MSC in polyQ soon led to the investigation of their therapeutic application in clinical studies. The clinical trials, mostly phase I/II, aiming at testing the safety and effectiveness of MSC in the treatment of polyQ disorders (completed and ongoing clinical trials— ClinicalTrials.gov Identifiers: NCT02728115, NCT03252535, NCT04219241, NCT03378414 for HD and NCT01360164, NCT01489267, NCT01649687, NCT02540655 for other polyQs/SCAs) are summarized in Table 2.

**Table 2:** Concise information on the outcomes obtained from clinical trials performed with MSCs in patients with PolyQ diseases.

Trial identification	Disease	Type of MSC	Route of administration	Outcomes
Dongmei et al. (2011)	SCA and MSA-C	UC-MSC	IT	ICARS and ADL (Katona et al. (2008)) scores significantly decreased 1 month after treatment. Unstable walking and standing, slow movement, fine motor disorders of the upper limbs and writing difficulties, and dysarthria were greatly improved except for 1 patient, who had no response. Ten cases (42%) remained stable for half a year or longer, while 14 cases (58%) had regressed to the status before the treatment within 1–14 months (an average of 3 months).
NCT01360164 Jin et al. (2013)	SCA1, SCA2, and SCA3	UC-MSC	IV and IT	After 1 year of treatment: 44% of the patients exhibited improved Berg Balance Scale (BBS) over baseline, and only 31% of the patients suffered from disease aggravation; 63% of the patients exhibited improved ICARS over baseline, and only 25% of the patients suffered from disease exacerbation.
Miao et al. (2015)	Several neurodegenerative disorders, including SCAs	UC-MSC	IT	Patients were followed-up for more than 1 year. No significant side effects were reported. Three of the 8 patients with SCAs (37.5%) showed motor function improvement 1 year after the treatments.
NCT01489267	SCA1	UC-MSC	IT	Unknown
NCT01649687 Tsai et al. (2017)	SCA3 or MSA-C	AD-MSC	IV	Patients were monitored for 1 year with no reported adverse events. They showed an initial trend toward brief improvement, followed by a slight stabilization and a progression of the disease in later stages. At 6 months follow-up, SARA scores were improved in 2 (33.3%), unaltered in 2 (33.3%), and worsened in 2 (33.3%) patients as compared with the baseline. At 1-year follow-up, SARA scores were improved in 1 (16.6%), unaltered in 3 (50%), and worsened in 2 (33.3%) patients as compared with the baseline.
NCT02540655	Cerebellar Ataxias	AD-MSC (Stemchymal^®^)	IV	Unknown.
NCT02728115	HD	Cellavita*	IV	Active, not recruiting
NCT03252535	HD	Cellavita	IV	Active, not recruiting
NCT04219241	HD	Cellavita	IV	This study is not yet open for participant recruitment.
NCT03378414	SCA1, SCA2, SCA3 and SCA6	UC-MSC	IV	Unknown

List of abbreviations: SCA, Spinocerebellar ataxia; MSA-C, and multiple system atrophy-cerebellar types; SCA1, Spinocerebellar ataxia type-1; SCA2, Spinocerebellar ataxia type-2; SCA3, Spinocerebellar ataxia type-3 or Machado-Joseph disease; SCA6, Spinocerebellar ataxia type-6; UC-MSC, umbilical cord-derived mesenchymal stromal cells; AD-MSC, adipose tissue-derived mesenchymal stromal cells; IT, Intrathecal injection; IV, Intravenous injections; ICARS, International Cooperative Ataxia Rating Scale; ADL, Activity of Daily Living; BBS, Berg Balance Scale. *Stem cells isolated from the dental pulp.

Data collected from four of these clinical trials have demonstrated that the therapeutic application of MSC is safe, does not produce severe side effects and might delay the progression of disease symptoms (Dongmei et al., 2011; Jin et al., 2013; Miao et al., 2015; Tsai et al., 2017). In one of these studies, fourteen patients diagnosed with SCA and ten with multiple system atrophy-cerebellar type C (MSA-C) were treated weekly with IT injections of UC-MSC at a dose of 1 × 10^6^/kg during four weeks (except for three patients who received two courses of treatment, all the other patients received only one course). Furthermore, the neuronal function and quality of daily life were assessed through the International Cooperative Ataxia Rating Scale (ICARS) and Activity of Daily Living Scale (ADL). Improvements in ICARS and ADL scores were observed after 1 month of treatment (Dongmei et al., 2011). In another study, Jin et al. (2013) demonstrated that MSC treatment could alleviate SCA symptoms for at least half a year. In this study, 16 patients diagnosed with SCA1, 2 or 3, received IV or IT injections of UC-MSC (first treatment: 4 × 10^7^ UC-MSC were infused iv.; following three treatments: 2 × 10^7^ UC-MSC were infused IV and simultaneously 2 × 10^7^ UC-MSC were infused by IT administration; all patients received only one-course treatment). Results showed that most patients obtained improved scores on the Berg Balance Scale (BBS), which evaluates patients’ balance, and ICARS at 3 and 6 months after treatment (Jin et al., 2013). A different clinical trial using UC-MSC was performed to evaluate the technical difficulties underlying IT UC-MSC injection *via* lumbar puncture and assess the effects of the cell in different neurodegenerative disorders, including eight SCA patients (Miao et al., 2015). The UC-MSC was injected 4–6 times between the L4 and L5 interspace within 5–7 days (single course of treatment). All patients were followed-up for more than 1 year for clinical status. Three of the eight patients showed motor function improvement after treatment. Recently, Tsai et al. (2017) intravenously administered (5–7 × 10^7^) allogeneic MSC derived from the adipose tissue (AD-MSC, single injection) into six patients with MJD and one with MSA, who showed a slight trend of improvement and stabilization of the disease according to the Scale for Assessment and Rating of Ataxia (SARA) and the sensory organization testing (SOT).

Importantly, no major side effects were reported, besides dizziness, headache, back pain in case of IT injections, or fever (Dongmei et al., 2011; Jin et al., 2013; Miao et al., 2015; Tsai et al., 2017). Consistently with these results, no adverse effects were reported so far in any of the pre-clinical studies. Moreover, in our study, we assessed for the levels of hepatic aminotransferases, which were not increased when compared to non-treated or WT mice, denoting the absence of liver toxicity in mice treated repeatedly with MSC (Oliveira Miranda et al., 2018).

Altogether, the studies reinforce the safety and tolerability of MSC. Furthermore, they demonstrate that treatment with allogenic UC-MSC may delay disease progression, improving motor performance, and patients’ quality of life without severe adverse reactions. However, the regression of some patients to the status before the treatment was reported (Dongmei et al., 2011; Jin et al., 2013; Miao et al., 2015; Tsai et al., 2017; summarized in Table 2). This was also following the need for performing several treatments suggested by some of the pre-clinical studies (Chang et al., 2011; Li et al., 2018; Oliveira Miranda et al., 2018). Nonetheless, in the clinical context, periodic MSC injections may not be feasible as they could lead to unwanted side effects. Indeed, some studies highlighted the long-term risk that could emerge from the repeated therapeutic transplantation of these cells, namely maldifferentiation, immunosuppression (increasing the risk of opportunistic infections), liver and lung accumulation, and malignant tumor growth promotion in patients with pre-existing malignancy (Sundin et al., 2006; Dierks et al., 2007; Kunter et al., 2007; Ning et al., 2008). On the other hand, as previously discussed, the local administration of MSC may cause brain damage if administered above a certain number (Rossignol et al., 2011). Therefore, it would be important to further clarify the mechanism involved in MSC therapeutic effects, uncovering new potential treatments that do not require the infusion of cells.

On the other hand, these clinical trials often mix patients with different conditions/stages of the disease and frequently include all disorders when reporting results, making reports not as informative as desired. Future clinical studies should focus not only on the feasibility of repeated treatments for sustained benefits but also on the appropriate timing, dosage and best method of injection to be used, while also performing longer follow-ups of all patients to evaluate treatment efficacy and safety, and with placebo-controls being used. For that purpose, established cohorts, well-defined and characterized must be performed so that clinical studies can be well-designed and as instructive as possible. An example of such a cohort has been implemented by the European Consortium ESMI (“European Spinocerebellar Ataxia Type 3/Machado-Joseph Disease Initiative,” Joint Programme on Neurodegenerative disorders), which put together eight European cohorts that over the last 4 years integrated all the existing data in a common database to allow for standardized and quality-controlled protocols in the forthcoming studies.

### Major Questions to be Addressed to Increase Clinical Benefits

From the clinical studies referred so far, we can conclude that the major weakness of the cellular therapies using MSC (or another adult/progenitor stem cell source) is that their effect is not prolonged throughout time. However, the cause of these transitory effects of MSC *in vivo* remains elusive. In our study, we could determine that MSC survival was limited in time after administration, which is consistent with other pre-clinical studies (as shown in Table 1). This may result from the inhospitable environment they face—the high oxidative stress of the damaged tissue, together with the low glucose availability—that leads MSC to choose an anerobic metabolic pathway, forcing cells to rapidly undergo apoptosis (Moya et al., 2018). Methods for increasing MSC fitness are described in the following section.

Another important question is whether MSC can effectively pass through the BBB upon intravenous injection. Several pre-clinical studies reported the finding of MSC in the central nervous system, including ours, as referred in Table 1. However, whether MSC crosses this barrier by mechanisms that are similar to leukocytes such as transmigration (Chamberlain et al., 2007), or whether this barrier needs to be impaired to let MSC pass-through remains undetermined. BBB leakage was already described for HD (Drouin-Ouellet et al., 2015). Due to the strong presence of inflammatory mechanisms in polyQ disorders, it can be speculated that this is also the case for the remaining polyQs. In accordance, we recently reported evidence that the BBB is damaged in MJD (Lobo et al., 2020). Still, there are no indications that the BBB opening is mandatory for MSC to reach the brain parenchyma. Studies accessing MSC biodistribution inside the nervous system in non-neurodegenerative rodents (wild type animals), should be performed to answer this intriguing question. In case MSC cannot cross the BBB when it is fully preserved, then other strategies should be explored such as the expression of specific receptors on the MSC surface that would allow their passage, or consider the use of MSC products (either naïve or engineered) that can effectively reach brain cells.

Finally, the prophylactic administration of this cellular treatment is also an important issue to be discussed. Since these disorders are monogenic and can be passed to the offspring, the patients can choose to know whether they carry the disease mutation before the manifestation of disease symptoms. Therefore, successful treatment could be implemented in pre-symptomatic patients. Accordingly, there is evidence that this would favor a later appearance of symptoms (Snyder et al., 2010; Li et al., 2018), supporting such early intervention as a feasible clinical approach for polyQs patients in the future. Nonetheless, Im and collaborators showed that MSC only modified the course of the disease when administered after its phenotypic expression in long-lived HD transgenic mice (Im et al., 2010). Therefore, the time window of delivery should be further investigated.

Overall, both pre-clinical and clinical studies show the therapeutic relevance of MSC for polyQ but also highlight the need to design better clinical approaches and more adjusted to the real biology and behavior of cells upon transplantation. Possible solutions are discussed in the following sections.

## Methods to Increase MSC’s Efficacy *In Vivo*

Strategies to overcome some of these shortcomings and improve MSC’s efficiency have been explored and will be described next, such as pre-conditioning of cells, the use of adjuvant factors, or the genetic enhancement of MSC. A concise review of that is given in Table 3.

**Table 3:** Summary of results using modified-MSCs in PolyQ diseases in comparison to naive cells—evidence from pre-clinical studies.

PolyQ	Study	Animal model	MSC’s source	Enhancement strategy	N° of cells injected	Site of injection	Time of injection	Outcomes compared with naïve cells
HD	Bantubungi et al. (2008); Edalatmanesh et al. (2011); Sadan et al. (2012)	QA-induced rat striatal model	Human BM-MSC isolated from healthy donors and HD patients differentiated into NTF^+^ secreting cells*	Pre-conditioning with supplemented medium	4.2 × 10^5^	Bilateral delivered at the lesion site	On the day of QA induction	NTF^+^ MSC led to less striatal volume loss and decreased T2 levels, as well as behavior improvements (reduced rotational behavior)
	Linares et al. (2016)	N171–82Q transgenic mice	Mouse BM-MSC	Pre-conditioning with Lithium and VPA	3 × 10^5^	Intranasal administration	8-week old mice (early symptomatic)	Pre-conditioned cells survived longer *in vivo* (60% more at 9 weeks after transplants), led to behavioral improvements, and reduced neuronal loss and aggregate formation
	Elbaz et al. (2019)	3NP-induced rat model	Rat BM-MSC	Combined administration of MSC and LER	1 × 10^6^	IV	1 h before 3-NP injections	Combined treatment decreased weight loss, and mortality rate to a greater extent; improved rotarod performance; decreased neuronal loss, inflammation, astroglia activation, and apoptosis
	Dey et al. (2010)	YAC 128 transgenic mice	BM-MSC	genetic modification to overexpress BDNF (MSC-BDNF) and NGF (MSC-NGF)	3 × 10^5^	Bilateral injections into the striata	4 Month old mice (post-symptomatic)	Both MSC-BDNF or MSC-NGF treated mice showed improved rotarod performances with MSC-BDNF having a greater effect and reduced neuronal loss
	Pollock et al. (2016)	YAC 128 transgenic Mice	human BM-MSC	genetic modification to overexpress BDNF (MSC-BDNF)	5 × 10^5^ per hemisphere	Bilateral injections into the striata	8.5-Month-old mice (post-symptomatic)	Only MSC-BDNF reduced mice anxiety levels; MSC-BDNF had a greater effect in decreasing striatal atrophy
		R6/2 transgenic mice	human BM-MSC	genetic modification to overexpress BDNF (MSC-BDNF)	5 × 10^5^ per hemisphere	Bilateral injections into the striata	7 Month old mice (symptomatic)	MSC-BDNF increased mice lifespan to a greater extent

List of abbreviations: HD, Huntington’s disease; QA, quinolinic acid; VPA, valproic acid; AD-MSC, adipose tissue-derived mesenchymal stromal cells; BM-MSC, Bone marrow-derived mesenchymal stromal; LER, lercanidipine; BDNF, Brain-derived neurotrophic factor; NTF, neurotrophic factors; NGF, Nerve growth factor IV, Intravenous injections; *NTF secreting cells produce and secrete large amounts of neurotrophic factors like BDNF and GDNF.

### Pre-conditioning of MSC

Knowing that the secretion of soluble and insoluble factors is an adaptive mechanism, which allows MSC to regulate intracellular stress and influence their surroundings, researchers have investigated whether the pre-conditioning of MSC could improve their therapeutic abilities. Pre-conditioning is nothing more than manipulating cells, so they perform a specific function or undergo a desirable differentiation pathway. Some studies showed that pre-conditioning of MSC using hypoxia, different culture conditions, biomolecules (cytokines and growth factors), or pharmacological compounds, may lead to greater MSC survival *in vivo* and enhanced regenerative and immunomodulatory effects (Ferreira et al., 2018; Noronha et al., 2019).

#### Pre-conditioning: Hypoxia

Oxygen tension under standard cell culture conditions (21%) is much higher than physiological oxygen tension in tissues, which can vary from 1% to 12% depending on vascularization and metabolic activity (Das et al., 2010). Notably, when we speak of hypoxia in the context of cell culture, usually it means that oxygen availability ranges from 0 to 10% to better mimic physiological conditions. MSC is usually found in tissues with low oxygen tension (1% to 7%), is naturally able to endure in *in vivo* hypoxic environments (Deschepper et al., 2011).

When cultured under hypoxic conditions, MSC increases their fitness to adapt to adverse circumstances which will make them better prepared to face inhospitable environments. Indeed, under hypoxia MSC changes their metabolism, increasing their glucose consumption, decreasing the production of oxygen species, and shortening telomeres (Das et al., 2010). In these conditions, MSC presents a greater proliferation rate and increased secretion of soluble factors without changing their multipotency, which improves MSC capacity to survive in damaged tissues upon transplantation (Das et al., 2010; Bader et al., 2015; Lee et al., 2017). Accordingly, Lee et al. (2017) showed that culturing MSC in hypoxic conditions leads to the up-regulation of Hypoxia-inducible factor (HIF)-α, which induces survival and proliferation rate.

Hence, UC-MSC pre-conditioned with hypoxia (MSC-h) showed to have neuroprotective, anti-apoptotic, and anti-inflammatory actions in a rat model of spinal cord injury. When compared with MSC cultured under standard conditions, MSC-h exhibited increased expression of neuroprotective trophic factors, such as hepatocyte growth factor (HGF), BDNF, and VEGF. Rats transplanted with MSC-h into the spinal cord immediately after lesion induction showed increased axonal preservation, even higher as compared to MSC not submitted to hypoxia. MSC-h also reduced the number of caspase-3 (apoptotic marker) positive cells, microglia, and macrophage infiltration (Zhilai et al., 2016).

Furthermore, Wang J. W. et al. (2017) demonstrated that hypoxia pre-conditioning enhanced the migration and integration of MSC upon transplantation into a rat model of cerebral ischemia, induced by cardiac arrest. These actions were linked with the activation of PI3K/AKT pathways and increased expression of HIF-1α and C-X-C chemokine receptor type 4. Plus, the IV injection of MSC-h was able to diminish neuronal death and inflammation in the cortex.

Even though these studies suggest hypoxia pre-conditioning may improve MSC neuroprotective effect and increase MSC survival after transplant, we found no studies that investigated the potential use of this approach in the treatment of neurodegenerative/polyQ disorders. Therefore, in the future efforts should be made to better explore this strategy.

#### Pre-conditioning: Biomolecules/Chemicals

Pre-conditioning of MSC with biomolecules such as cytokines, growth factors, or hormones can improve MSC survival and efficiency. Indeed, Sadan and collaborators showed that MSC that were pre-conditioned in media supplemented with epidermal growth factor, human basic FGF, cyclic adenosine monophosphate, human neuregulin1-*β*1, and platelet-derived growth factor (Sadan et al., 2008, 2009) differentiated into NTF secreting cells (NTF+) cells, which could secrete high levels of BDNF and glial cell line-derived neurotrophic factor (GDNF). NTF+ MSC could improve both the neuropathology and behavior patterns in the QA rat model of HD (Sadan et al., 2012). QA-injected rats transplanted with NTF+ MSC lost less striatal volume than rats transplanted with naïve MSC. Moreover, NTF+ MSC decreased T2 values when compared with PBS-treated QA rats, as detected by MRI acquisitions. Phenotypically, NTF+ cells were more efficient in reducing rotational behavior than naïve MSC in QA-injected rats, thus suggesting these cells to be more efficient.

In a recent study, the authors assessed the impact of culturing MSC with different sera. MSC was cultured in medium with 10% of the following sera: fetal bovine serum, serum from healthy controls (NS-MSC), or serum from stroke patients (SS-MSC). Interestingly, SS-MSC had a higher proliferation rate and lower senescence as well as increased expression of VEGF, GDNF, and FGF. Additionally, SS-MSC promoted neurogenesis and angiogenesis in stroke rat models, leading to improved behavioral performance (Moon et al., 2018).

Alternatively, MSC can be primed using pharmacological or chemical agents. Linares et al. (2016) investigated whether pre-conditioning MSC with lithium and valproic acid (VPA) could enhance their therapeutic effect. Lithium and VPA are mood stabilizers known to exert neuroprotective effects (Chiu et al., 2013) and pre-conditioning of MSC with these factors led to an increase in the expression of genes involved in trophic effects, as well as in pro-survival and immunomodulatory pathways. Accordingly, N171–82Q transgenic HD mice were treated with an intranasal administration of MSC or MSC pre-conditioned with Lithium and VPA. The transplanted cells migrated into the brain, with pre-conditioned MSC surviving for a longer period. Moreover, mice treated with pre-conditioned MSC showed a greater amelioration of motor and behavior performance, decreased neuronal death, and reduction of huntingtin aggregates in the striatum (Linares et al., 2016). This strategy could be used in other polyQ diseases where Lithium and VPA showed promising results (Saute et al., 2014; Esteves et al., 2015; Lei et al., 2016; Lopes-Ramos et al., 2016).

Therefore, though pre-conditioning needs to be further explored, the results obtained so far seem promising, which opens good prospects for the near future.

### Combined Treatment With Pharmacological Agents

A less common strategy recently evaluated involved the use of pharmacological agents in combination with MSC to treat polyQ disorders. Elbaz et al. (2019) showed that the combined administration of Lercanidipine (LER), an antihypertensive drug, and MSC boosted their therapeutic efficiency in a rat model for HD. LER is a vasoselective dihydropyridine calcium channel blocker that can modulate calcium levels and, therefore may be able to influence the calcineurin (CaN)/nuclear factor of activated T cells c4 (NFATc4) pathway, which is deregulated in neurodegenerative disorders (Sompol and Norris, 2018). Treatment with LER, BM-MSC, or a combination of LER and BM-MSC was given to 3-NP rats. The combined treatment with LER and BM-MSC had the most promising results by leading to greater amelioration of motor and behavioral performance. Moreover, the levels of BDNF, forkhead box P3, Wnt, and *β*-catenin increased in the striatum, along with a decrease of CaN, tumor necrosis factor-α, and NFATc4 protein expression and the Bax/B-cell lymphoma 2. Their results suggest that this combined therapy can promote neuroprotective effects by, at least in part, suppressing Ca^2^+/CaN/NFATc4 and Wnt/*β*-catenin signaling pathways activation, which is dysregulated in the 3-NP rats as well as in other neurodegenerative disorders (Sompol and Norris, 2018).

Hence, this study shows how a combination of factors can produce a synergistic effect, so it would be interesting to further investigate the neuroprotective potential of therapies combining different drugs with MSC.

### Genetically Engineered MSC

Another strategy that has been studied to enhance MSC’s therapeutic efficiency is the production of genetically engineered MSC that can be used as delivery vehicles (Park et al., 2015). For the treatment of neurodegenerative diseases, the most common approaches include the use of MSC that were either virally or non-virally modified to overexpress trophic factors known to have neuroprotective actions (Huang et al., 2012; Deng et al., 2016).

Concerning polyQ disorders, only two studies are available in the literature. The safety and efficacy of genetically engineered MSC have been tested in HD animal models. In 2010, BM-MSC were genetically engineered to over-express BDNF (MSC-BDNF) and/or NGF (MSC-NGF), thus allowing these cells to deliver these factors in higher amounts than normal MSC (Dey et al., 2010). YAC128 transgenic mice were treated with MSC-BDNF, MSC-NGF, or with both MSC-BDNF and MSC-NGF. MSC was administered through bilateral injections into the striatum. From all the tested groups, YAC128 transgenic mice transplanted with MSC-BDNF exhibited the best outcomes, showing the least neuronal loss and the highest latencies to fall values in rotarod.

Another study demonstrated that human MSC modified by lentiviral transduction to overexpress BDNF (MSC-BDNF) could ameliorate HD symptoms in two different animal models, YAC128 and R6/2 transgenic mice in a higher extent (Pollock et al., 2016). The intrastriatal transplantation of MSC-BDNF decreased striatal atrophy and reduced anxiety levels in YAC128 mice. Additionally, MSC-BDNF administration promoted neurogenesis-like activity and led to an increase in mice lifespan. Notably, mice treated with MSC-BDNF had overall better outcomes than those treated with non-modified MSC.

Despite the few studies using genetically engineered MSC to treat polyQ disorders, this type of approach has been further explored in other neurodegenerative diseases, namely in AD and PD, also with promising results (Li et al., 2008; Moloney et al., 2010; Ren et al., 2013; Hoban et al., 2015).

Importantly, since neurodegenerative disorders have common pathogenic mechanisms, current evidence obtained from these studies indicate that genetically modified MSC to overexpress various trophic factors is strong therapeutic candidates for several neurodegenerative diseases, including polyQ disorders.

## Cellular-Free Approaches

### The Success of Studies Using MSC’s Secretome in PolyQ/Other Neurodegenerative Disorders

Several studies detected reduced numbers of MSCs in the targeting tissues suggesting that MSCs can also act through paracrine mechanisms. Conditioned medium (CM) of MSC (CM-MSC), i.e., MSC’s secretome, can reproduce the therapeutic effects obtained with MSC treatment in several neurological diseases.

Bai et al. (2012) demonstrated that CM-MSC mitigates functional abnormalities in a mouse model of multiple sclerosis by promoting oligodendrocyte and neuron development. Suto et al. (2016) showed that injections of MSC released factors, such as HGF, improved motor coordination in an SCA-1-Knock-in mouse model. Indeed, the administration of CM-MSC was able to protect axons and myelin of spinal motor neurons (Suto et al., 2016). Importantly, such improvements were similar to those obtained in previous work using the same model of disease in which animals were treated with MSC (Mieda et al., 2016).

An antibody-based protein array analysis and ELISA showed that, in addition to HGF, IGF-1, VEGF, and transforming growth factor (TGF)-β-1 are present in the CM. However, the combination of these factors is not as effective as treatment with CM in promoting neuronal survival and neurite outgrowth, which suggests that CM contains other complementary key factors (Nakano et al., 2010). The secretion of BDNF and NGF by MSC has also been demonstrated to promote neuronal/glial survival and neurogenesis (Crigler et al., 2006). Likewise, the beneficial effect of MSC may also be mediated by secretion of GDNF, basic FGF-2, CNTF, angiopoietin-1, and neurotrophin-3 (Chen et al., 2005; Onda et al., 2008). Besides, the extracellular matrix proteins, including collagen, fibronectin, and laminin (Hidalgo-Bastida and Cartmell, 2010) provide physical guidance, support neurogenesis, and may exert trophic activity themselves (Maltman et al., 2011).

Recently, Ebrahimi et al. (2018) demonstrated that CM collected from UC-MSC protect PC12 cells against superoxide-induced oxidative stress, increasing cell viability, and neurite outgrowth. GDNF and VEGF were detected in CM of UC-MSC and were linked to the neuroprotective effects observed.

Moreover, it has been demonstrated that both human adipose-derived MSC and their CM could reduce neuronal cell damage in a model of glutamate excitotoxicity, a mechanism that plays a role in the pathogenesis of neurodegenerative diseases. Indeed, co-cultures with MSC using a transwell system or addition of CM similarly promoted axonal regeneration, energy metabolism improvements, and prevented apoptosis of cortical neurons (Hao et al., 2014). In another study, Miranda et al. (2011) showed that in a transwell system that immortalized BM-MSC could stimulate neurite outgrowth in a neurotrophin-dependent manner including BDNF secretion in Twitcher-derived DRG neurons, an *in vitro* model of Krabbe’s disease. Moreover, the administration of exogenous BDNF to the sciatic nerve of Twitcher mice through BDNF-delivering osmotic pumps promoted morphometric ameliorations, suggesting BDNF as a promising candidate to be used in combination with BM-MSC treatment (Miranda et al., 2011). Nevertheless, simultaneous transplantation of BM-MSC and treatment with an antagonist of TrK receptors in a model of sciatic nerve crush completely reversed the inhibitory effect of the antagonist, suggesting that other mechanisms may be compensating the action of neurotrophins blocked by the antagonist.

A recent study has shown that CM-MSC promoted neuroprotective activities in *in vitro* PD models by increasing the viability of both rat and human dopaminergic neurons exposed to a neurotoxic insult (Parga et al., 2018). Additionally, the administration of MSC secretome through an injection into the substantia nigra and striatum in a PD mouse model led to an increase of dopaminergic neurons and neuronal terminals in those brain regions, which likely mediated the observed temporary motor performance improvements. Also, their findings suggest that the beneficial effects of human MSC secretome correlated with the increased presence of several neuroregulatory molecules, such as GDNF, BDNF, interleukin-6 (IL-6), VEGF, pigment epithelium-derived factor (PEDF), cystatin C, glia-derived nexin (GDN) and galectin-1 (Teixeira et al., 2017).

Alternatively, MSC potent anti-inflammatory and immunoregulatory effects might play a central role in tissue regeneration. For instance, MSC are suggested to inhibit T and B cell proliferation (Di Nicola et al., 2002; Franquesa et al., 2012), natural killer cytotoxicity (Spaggiari et al., 2008), and monocyte differentiation and maturation into dendritic cells (Ivanova-Todorova et al., 2009). These capacities have been linked to the release of molecules like indoleamine 2,3-dioxygenase (IDO), prostaglandin E2, interleukin-10 (IL-10), human leukocyte antigen-G, TGF-β, and HGF (Dorronsoro et al., 2013). It has been demonstrated that the systemic administration of CM derived from amniotic membrane MSC ameliorates motor dysfunctions, brain pathology, and decreased microglial activation in HD animal model (Giampà et al., 2019).

In summary, the bioactive molecules released by MSC can exert angiogenic, immunomodulatory, neurogenic, neuroprotective, and anti-apoptotic effects. Therefore, MSC’s secretome seems to improve symptoms of several neurodegenerative disorders, including polyQs, in a paracrine-mediated manner, representing an alternative to cell therapies.

### Extracellular Vesicles: Important Mediators of MSC Communication With Host Cells and Their Environment

**Figure 3:**
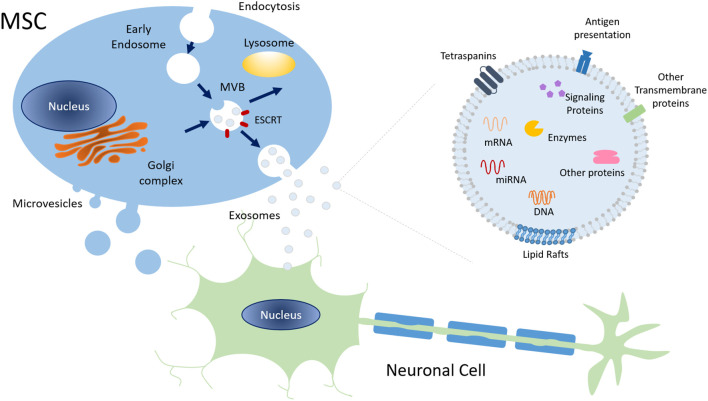
Schematic representation of exosome biogenesis and molecular composition. Exosomes have an endocytic origin and are released to the extracellular milieu upon the fusion of MVB with the plasma membrane. Other MVB may fuse with lysosomes leading to their degradation. Overall, exosomes surface markers, cytoskeleton, and cellular metabolism proteins are common to those of the cell of origin. Plus, exosomes also include signaling proteins, DNA, several types of RNA, and lipids. Abbreviations: ESCRT, Endosomal Sorting Complex Required for Transport; MVB, Multivesicular bodies; mRNA, messenger RNA, miRNA, microRNA.

MSC are known to release EVs, a group of heterogeneous membrane-limited structures secreted by almost all cell types. Exosomes are the most studied subpopulation of EVs that can are divided by intracellular origin, size, and cargo. EVs are recognized as important mediators of intercellular cross-talk communication, being involved in both physiological and pathological processes (Thery et al., 2002; Zöller, 2009; Colombo et al., 2014). In fact, after being released into the extracellular space, EVs can be internalized by recipient cells and transfer their content. Then, their cargo may either be degraded or mediate various signaling functions, modifying cellular fate, function, and/or plasticity (Figure 3; Mulcahy et al., 2014; Abels and Breakefield, 2016).

Depending on their subpopulation, the cell type of origin, and specific environmental challenges (for example stress conditions), EVs biomolecular content will change, bearing different lipids, proteins, and nucleic acids. To standardize the field, MISEV guidelines were created by the International Society of Extracellular vesicles (ISEV; Thery et al., 2018). Moreover, a harmonization criterion for MSC-EVs was created by four societies, ISEV, Society for Clinical Research and Translation of Extracellular Vesicles Singapore (SOCRATES), International Society for Cell and Gene Therapy (ISCT), and International Society of Blood Transfusion (ISBT), to boost MSC-EVs applications in clinical settings (Witwer et al., 2019).

Given EVs’ physiological importance and role in brain disorders, their potential therapeutic use has been explored over the last decades. A comprehensive review can be found in Rufino-Ramos et al. (2017).

#### EVs Derived From MSC

The general morphological characteristics of EVs derived from MSC are common with EVs from other sources, yet they possess some distinctive features. Besides common surface markers, like CD9, CD63, and CD81, MSC-derived EVs also possess specific MSC integrins and adhesive molecules, including CD29, CD44, CD90, and CD73 (Lai et al., 2012). Their combination defines tropism to specific tissues.

EVs derived from MSC encapsulate mRNAs, miRNAs, and proteins related to multiple signaling pathways, namely cell cycle, proliferation, differentiation, and self-renewal (Lai et al., 2010; Kim et al., 2012). However, as aforementioned, extracellular conditions can affect their composition (Xin et al., 2014). Indeed, Lai et al. (2012) demonstrated that MSC-derived EVs obtained from CM from three different batches of the same cells had only 154 proteins in common. Furthermore, pre-conditioning MSC with other cells and/or exposure to disease insults may change exosomes’ secretion profile (Kim et al., 2007; Sano et al., 2014; Cui et al., 2018).

Over the last decade, EVs derived from MSC have become promising therapeutic tools as researchers hypothesized that they may be the main paracrine effectors of MSC. In 2010, the therapeutic potential of MSC-derived EVs was tested in a mouse model of ischemia (Lai et al., 2010). Since then, EVs have been investigated in several other disease models, such as cardiovascular disorders (Wang N. et al., 2017), kidney injury (Aghajani Nargesi et al., 2017; Farzamfar et al., 2019), immune diseases (Lai et al., 2018, 2019), tumor growth (Wu et al., 2013; Zhang et al., 2017) and neurological diseases (Wang et al., 2018; Gorabi et al., 2019), obtaining encouraging results (Yu et al., 2014).

MSC-EVs have been shown to exert similar functions to those of the progenitor cells, participating in tissue repair and regeneration, immune system modulation, and inflammatory suppression in neurodegenerative disorders (Pashoutan Sarvar et al., 2016). Xin et al. (2013) performed a proof-of-concept study providing evidence that treatment with MSC-derived EVs alone could promote functional recovery, neurogenesis, and neurovascular remodeling in rats subjected to middle cerebral artery occlusion, a stroke model. Interestingly, the outcomes were similar to those obtained with systemic administration of MSC. In a previous study, the same group had found that MSC was able to promote neurite outgrowth and functional recovery partially by the transference of miRNA 133b (miR-133b) *via* exosomes (Xin et al., 2012). Levels of miR-133b were increased in the ipsilateral hemisphere of the stroke rat model after treatment with MSC. Plus, *in vitro* exposure of MSC to damaged brain tissue led to the enrichment of miR-133b in cells and their exosomes. Finally, when primary neurons co-cultured with astrocytes were treated with these exosomes, miR-133b levels were found to be increased. Moreover, this effect was lost in astrocytes treated with a miR-133b inhibitor. In this way, Xin et al. (2012) showed that exosomes play a role in MSC communication with brain parenchymal cells, mediating the transfer of bioactive molecules like miR-133b. In a different context, EVs isolated from adipose-derived stromal cells (AD-MSC) exerted a neuroprotective effect in an *in vitro* model of amyotrophic lateral sclerosis (ALS). Upon an oxidative insult, the motoneuron-like NSC-34 cells expressing ALS mutations were treated with exosomes, which showed the capacity to protect ALS motoneurons from oxidative stress, increasing their viability (Bonafede et al., 2016).

In line with those results, in another study, it was demonstrated that both MSC and MSC-derived EVs were able to protect hippocampal neurons from oxidative stress and synapse damage induced by amyloid-β peptide (AβO) upon IC delivery (de Godoy et al., 2018). These authors also unveiled that such neuroprotective action could be explained by MSC’s ability to internalize and degrade AβO, secret EVs, as well as trophic and anti-inflammatory cytokines. EVs were shown to contain an antioxidant enzyme, catalase, which contributed to the decrease of oxidative stress. Recently, Reza-Zaldivar et al. (2019) also reported that both MSC and MSC-derived EVs were similarly able to promote neurogenesis and ameliorate cognitive impairments caused by β amyloid 1–42 aggregates, an established Alzheimer’s disease animal model.

Hypoxia conditions were shown to increase mir-21 levels in MSC cells and their derived EVs. By systemically injecting EVs in Transgenic APP/PS1, it has been shown that EVs produced during hypoxia could reach the brain and increase the levels of mir-21. By increasing the levels of mir-21 in the brain, learning and memory capabilities were restored while plaque deposition and Aβ levels were decreased. These findings substantiate the therapeutic potential of MSC-EVs in Alzheimer’s disease context (Cui et al., 2018).

MSC can ultimately be genetically modified to enhance EVs production that specifically targets the brain and promote cargo delivery in neurons. Kojima et al. (2018) engineered MSC to: (1) boost EVs production by acting in exosomes biogenesis; (2) package mRNA catalase inside EVs; and (3) express at EVs surface the RVG peptide that targets the brain and connexin 43 to enhance information transference in the target cells. This study showed that engineered cells can produce modified EVs able to deliver therapeutic cargo to the brain in a PD model (Kojima et al., 2018).

One of the major drawbacks of using MSC-EVs for brain therapy is the low number of vesicles that cross BBB and reach the brain. To overcome this, a recent study engineered MSC derived vesicles with magnetic properties that reach the brain after application of a magnetic field (Kim et al., 2020). Moreover, stimuli with magnetic particles increase growth factors expression in MSC-derived vesicles. These magnetic vesicles were administered through a systemic injection in middle-cerebral-artery-occlusion (MCAO)-induced rats promoting anti-inflammatory response, angiogenesis, and anti-apoptosis in the ischemic brain lesion leading to an improvement in the motor function.

Altogether, these studies support the idea that EVs derived from MSC may be an important player in the paracrine effect of MSC and, therefore, a potential therapeutic agent for the treatment of polyQ diseases.

## How to Standardize MSC or MSC’s Effectors?

As aforementioned, multiple extrinsic factors including tissue source, culture methods, passage number, and oxygen concentration can significantly interfere with MSC innate functional potential (Hagmann et al., 2013; Lee et al., 2013; Heathman et al., 2015; Elahi et al., 2016). To comply with Good Manufacturing Practices (GMP) and current Good Tissue Practices, certain aspects of MSC or MSC’s effector’s production like: (1) the method of isolation; (2) culture medium, serum and supplements used; (3) cell seeding density; and (4) devices of expansion, must be optimized and standardized, thus ensuring consistent production, efficacy, and safety (Rojewski et al., 2013; Sensebé et al., 2013; Sharma et al., 2014).

Another important aspect that needs further clarification is the cryopreservation procedure as no consensus on whether the use of freshly cultured or freeze-thawed MSC is more advantageous. Multiple studies are showing that thawed MSC/MSC products have reduced therapeutic capabilities (François et al., 2012; Chinnadurai et al., 2016, 2018) and, contrarily, some studies demonstrate that both thawed and fresh MSC have similar functionalities (Cruz et al., 2015; Bárcia et al., 2017; Tan et al., 2019). One explanation for this disparity may be the temperature fluctuation under frozen conditions (Chabot et al., 2017).

Given the heterogeneity of practices and outcomes among different laboratories, both in pre-clinic and clinic grades, in 2016 ISCT issued a position statement urging the need to identify MSC functional markers of potency and implement assays for the measurement of such markers. This is a fundamental step that could help in predicting and improving the efficiency of MSC related therapies, meeting the requirements of Regulatory Authorities for extensive quality-control protocols for advanced clinical studies and registrations (Galipeau et al., 2016). Nevertheless, MSC has complex mechanisms of action that are not yet completely understood, which hinders the determination of which biological proprieties of MSC are more relevant to assess (Galipeau and Krampera, 2015). Ideally, multiple complementary assays (matrix assay) should be developed to characterize several attributes of MSC/MSC’s effectors that may be important for each therapeutic purpose (Galipeau et al., 2016).

Regarding studies that use MSC/MSC’s effectors as a therapeutic agent for neurodegenerative disorders, in addition to current characterization criteria, which include the presence and/or absence of specific markers and their differentiation potency (Dominici et al., 2006), it is essential to establish assays for the analysis of their immunomodulatory capability and senescence status, especially in clinical settings. A summary of the assays that could be used to standardize MSC/MSC’s effectors is given in Table 4.

**Table 4:** Summary of assays that can be used to standardize MSC/MSC’s effectors.

Study	Assayed Parameters	Assayed description/outcomes
Tarte et al. (2010)	MSC senescence	Evaluation of the expression of key genes of senescence pathways (myc, p21, p53, p16^ink4^, hTERT) to guarantee MSC fitness in later passages.
Boregowda et al. (2016)	Angiogenic, anti-inflammatory and immunomodulatory potential	Clinical Indications Prediction scale that assesses MSC potential in accordance to the expression levels of the transfection factor TWIST1: ↓ levels of TWIST1–↑ anti-inflammatory and immunomodulatory potential; ↑ levels of TWIST1–↑ angiogenic potential
Chinnadurai et al. (2019)	Immunomodulatory potential	Assay matrix approach that assesses MSC immunoinhibitory potential upon of MSC-PBMC co-culture: ↓ TNF-α, IFNγ, IL-13, IL-5, IL-2R, CCL3, and CCL4 cytokine levels in the secretome–↑ suppression of T-cells; ↑ VEGF, IFNα, CXCL10, GCSF, CXCL9, IL-7, and CCL2 levels in the secretome–↑ suppression of T-cells
Chinnadurai et al. (2019)	Immunomodulatory potential	Assay matrix approach that assesses MSC immunoinhibitory potential based on the phosphorylation of STAT in MSC after contact with the secretome of MSC-PBMC co-culture: ↓ phosphorylation of STAT1 and STAT3–↑ suppression of T-cells

*List of abbreviations: CCL2, monocyte chemoattractant protein-1; CCL3, macrophage inflammatory protein-1 α; CCL4, Chemokine C-C motif ligands 4; CXCL, C-X-C Motif Chemokine Ligand; IL, interleukin; IFN, Interferon; GCSF, Granulocyte colony-stimulating factor; MSC, Mesenchymal stromal cells; PBMC, peripheral blood mononuclear cells; STAT, signal transducer and activator of transcription; TNF-α, Tumor Necrosis Factor-α; VEGF, Vascular endothelial growth factor*.

It has been demonstrated that after multiple passages, MSC enters senescence, and cells show loss of differentiation potential, different secretion profiles, and telomere shortening (Wagner et al., 2008). Thus, sensitive evaluation of key genes and/or molecules involved in senescence pathways such as myc, p21, p53, p16ink4, hTERT is essential to guarantee the genomic stability of MSC in long-term culture (Tarte et al., 2010).

In 2018, Chinnadurai et al. (2018) proposed for the first time a dual assay matrix approach that combined secretome analysis and quantitative RNA-based array to characterize MSC crosstalk interaction with peripheral blood mononuclear cells (PBMC) upon co-culture. In this study the authors identified cytokines and chemokines whose increased or decreased expression directly correlated with MSC ability to suppress PMBC proliferation, reporting on MSC functionality to modulate the secretome immune response (Chinnadurai et al., 2018). Recently, the same group developed another assay matrix approach based on the phosphorylation of signal transducer and activator of transcription (STAT) of MSC after interaction with MSC-PBMC co-culture secretome. This informs on MSC functionality to sense and modulate the environment. The authors reported that STAT1 and STAT3 phosphorylation correlated with MSC immunoinhibitory ability (Chinnadurai et al., 2019). Nevertheless, depending on the disease, instead of PBMCs, the use of immune effector cells directly involved in the pathogenic mechanisms of the diseases may be more suitable and preferable.

Furthermore, Boregowda et al. (2016) developed a Clinical Indications Prediction (CLIP) scale for different diseases that predicts the therapeutic efficacy of populations of MSC from different donors, based on the expression levels of the transfection factor TWIST1. MSC expressing high levels of TWIST1 have greater angiogenic potential whereas low TWIST1 expressing populations are predicted to have higher anti-inflammatory and immunomodulatory capabilities.

Nonetheless, these potency assays still bear limitations, not providing complete predictive guidance. To complement these strategies in the future, it would also be important to study patient clinical parameters, such as the stage of the disease, co-morbidities, and age, among others, which could help predict patient responsiveness to treatment. Additionally, donors’ age, gender, and clinical status should also be carefully considered. As different studies offer contradictory information on the impact of such parameters (Stolzing et al., 2008; Andrzejewska et al., 2019), a greater understanding of how these characteristics may correlate with MSC proliferation, multipotency, and efficiency is needed.

We expect that further research may shed light on MSC heterogeneity and their highly complex mechanisms of action, thus facilitating the optimization and standardization of MSC/MSC effector processes, leading to higher quality-control protocols along with better and more consistent therapeutic outcomes.

## Conclusion: MSC—Do They Have A Future in PolyQ Clinical Therapies?

MSC have gathered considerable interest among the scientific community and their application to neurodegenerative disorders has not been an exception. It has been reported that the therapeutic application of MSC can produce encouraging results as they can promote tissue regeneration and cell turnover by: (i) increasing cell survival; (ii) inducing neurogenesis; (iii) inhibiting apoptosis; and (iv) modulating inflammation, among others, mainly through paracrine mechanisms. Further supporting their therapeutic potential, clinical trials have demonstrated that MSC are safe and may produce beneficial results in different neurodegenerative disorders. In polyQ disorders, patients enrolled in clinical trials showed a delay in the progression of disease symptoms. However, a considerable percentage of patients were reported to regress to their status before transplantation, indicating that MSC beneficial effects must be transient. On the other hand, the lack of appropriate animal models that could better mimic these human diseases is another limiting issue, as this hardens result interpretation.

Meanwhile, several strategies have emerged that aim at increasing either MSC survival or their efficacy *in vivo*. Pre-conditioning with hypoxic conditions or biomolecules, a combination of MSC with other pharmacological agents, or genetic modification of cells are some of these possible strategies. On the other hand, due to the strong paracrine properties of MSC, cell-free approaches using MSC secretome or MSC-derived EVs have naturally become a motif of interest in the case of neurological disorders, as they take advantages of the paracrine neuroprotective effect of MSC. Finally, it would be important to not only standardize procedures regarding MSC-derived therapies but also to uncover the role that MSC exert in polyQ models. Pinpointing possible MSC effects will certainly open avenues for triggering novel therapeutic targets for this group of neurodegenerative disorders.

As a conclusion, the final goal may now be to implement more standardized studies to discover a promising cell or cell-free based therapeutic strategy amenable to systemic delivery and enabling stably reaching the brain. In our point of view, this strategy would mean an add-on value in the clinics as it can be easily implemented due to its safety and minimally invasive character.

## Author Contributions

IB: discussion and article writing, and figure design. AM, TS, and JB: article writing. DR-R: article writing and figure design. LP: article discussion and reviewing. CM: article outline, discussion and article writing, and reviewing. All authors contributed to the article and approved the submitted version.

## Conflict of Interest

The authors declare that the research was conducted in the absence of any commercial or financial relationships that could be construed as a potential conflict of interest.
